# Religiosity of adults on the autism spectrum: a cognitive and empirical analysis

**DOI:** 10.3389/fpsyt.2025.1594692

**Published:** 2025-07-01

**Authors:** Agnieszka Ewa Burnos, Gabriela Kopacz

**Affiliations:** Faculty of Psychology, University of Warsaw, Warsaw, Poland

**Keywords:** autism spectrum disorder, religion, religiosity, theory of mind, autism

## Abstract

This article presents a narrative theoretical and empirical review of religiosity in adults on the autism spectrum. Religiosity is defined as an individual set of beliefs and practices proposed by a religious institution or group. This topic is critical for better understanding the religious and spiritual needs of autistic individuals, as well as the barriers they may face in developing and practicing religiosity. Theoretical accounts of the relationship between the social and cognitive characteristics of individuals on the autism spectrum and their religious attitudes and behaviors are examined. These include theory of mind, weak central coherence, executive function deficits, restricted interests, need for predictability, cognitive rigidity, and the broken mirror hypothesis. Alongside these conceptual frameworks, the article reviews findings from nine empirical studies. The emerging picture of religiosity among autistic adults is complex and marked by inconsistency. The central hypothesis—that impairments in mentalizing reduce religiosity—has not been unequivocally supported by empirical evidence. Similarly, results regarding the overall level of religiosity and representations of God in autistic versus neurotypical individuals are inconclusive. The article offers a synthetic overview of existing hypotheses and provides recommendations for the design of future research in this area.

## 1 Introduction

Religion has accompanied humanity for thousands of years. The methods and subjects of religious studies have changed over time, much like the understanding of autism, which has a shorter presence in scientific and societal discourse. One of the more recent research approaches to the study of religiosity is the cognitive science of religion (CSR). This interdisciplinary framework—referred to in Polish literature as either cognitive religious studies or the cognitive science of religion—emerged from the “cognitive revolution” of the 1950s. According to the CSR perspective, religious phenomena, behaviors, and beliefs are shaped by cognitive processes (1, 2). Despite emerging criticism of CSR’s reductionist assumptions (3–7), the model is widely applied to describing the specific characteristics of religiosity in individuals on the autism spectrum. It offers potential explanations for the hypothesized lower-than-average levels of religiosity in this population by drawing on the theory of mind (ToM) and related hypotheses (8–11). Other mechanisms found in the literature that aim to explain the relationship between autism and religiosity include weak central coherence, restricted interests (8), cognitive rigidity (12), and mirror neurons (13).

This paper presents a review of the theoretical foundations identified in the literature that relate religiosity to autism, as well as empirical studies that examine these assumptions. The aim of this paper is also to describe the specific nature of religiosity among adults on the autism spectrum. The focus is on adult religiosity, which is generally perceived as more stable than that observed during earlier, more dynamic developmental periods (14–16). A separate review of religiosity among adolescents with autism spectrum disorder has been conducted by Hayat et al. (17).

The scope of religiosity among individuals with autism spectrum disorder is conceptually ambiguous due to the broad meanings of the terms involved. Both “autism,” which has just over a century of history, and “religiosity” require clarification for the purposes of this article. In the main body of this work, the terms are used as defined by the original authors.

## 2 Historical context of the autism spectrum

Autism has come to be understood in many ways and is associated with numerous related terms used across social, medical, and scientific contexts. It is worth noting that while the term “autism” and its derivatives only emerged in the 20th century, societies prior to this also recognized specific patterns of social and cognitive development among their members. Evidence of this can be found in traditional folktales featuring children who “failed to meet their parents’ expectations” or were “misfits” (18).

The term “autism” was first introduced in the early 20th century. In 1910, Swiss psychiatrist Eugen Bleuler used the term “autistic thinking” to describe the thought patterns of some of his patients diagnosed with schizophrenia. Fifteen years later, Russian child psychiatrist Grunya Sukhareva used the term “autistic tendencies” to describe six of her patients. The work of these physicians only gained recognition in the scientific and medical communities posthumously (19). In the 1940s, publications by American psychiatrist Leo Kanner (1943) and Austrian pediatrician Hans Asperger (1944) established autism as a significant developmental disorder. Kanner’s description of early infantile autism included a triad of symptoms: difficulty forming relationships with others, repetitive behavior patterns, and impaired verbal communication. Kanner’s publication (1943) quickly gained influence in medical circles and helped shape diagnostic criteria for autism. Asperger’s observations on autistic psychopathy were later popularized in the 1980s by Lorna Wing (20). Childhood autism was first classified as a separate disorder within the pervasive developmental disorders category in DSM-III (21). Asperger’s syndrome was introduced in DSM-IV as a subtype of autism (22) and also in ICD-10 (23). It was differentiated from childhood autism by the absence of significant delays in language or cognitive development.

The understanding of autism has undergone significant change in recent decades (19). A shift in conceptualization was introduced in DSM-5 (24) and ICD-11 (25), which abandoned the division into distinct diagnostic entities within pervasive developmental disorders, including the separate classification of Asperger’s syndrome. Instead, a model of autism spectrum disorders (ASD) was proposed, differentiated by the severity of symptoms across dimensions. DSM-5 (24) specifies three levels of severity for ASD based on the amount of support needed by the individual. These levels are determined by the degree of difficulty in social communication and the presence of restricted, repetitive patterns of behavior. ICD-11 (25) allows for numerous symptom combinations, reflecting the high heterogeneity of autism presentation. Critics argue that increasing diagnostic heterogeneity distances ASD criteria from the concept of a measurable neurodevelopmental or behavioral disorder, presenting instead a complex picture of internal subjective experience (26).

The term “high-functioning autism” (HFA) is a translation of the English term and lacks clear boundaries. It is often used interchangeably with Asperger’s syndrome, which is no longer a formal diagnostic category (27, 28). Attempts have been made to distinguish between the two (29, 30). According to the Encyclopedia of Autism Spectrum Disorders, HFA describes individuals on the autism spectrum whose cognitive or linguistic abilities are at least average for their age. Although there are no standardized clinical or scholarly criteria for the term, it remains widely used in research. It is noted that this subgroup is overrepresented in studies (27). The term closely resembles a DSM-5 (24) category indicating individuals requiring the lowest level of support. The label is not universally accepted in Poland, where Ewa Pisula (31) recommends the term “intellectually well-functioning individuals with autism.” Scholars critique the HFA label, noting that it encompasses individuals with significant variation in adaptive functioning, which does not always align with cognitive ability (27, 32). The ambiguity of the category and evolving understanding of autism led Frith (33) to question whether a clear boundary can be drawn between autism spectrum disorders and typical personality differences. This aligns with current diagnostic perspectives that conceptualize autism more as a continuum than as distinct categories.

## 3 Foundations of religiosity

Although religion has been part of human life for millennia, the psychology of religion as a research field was only established in the 20th century. Starbuck was the first to use the term “psychology of religion” in his book presenting survey-based research on religious conversion (34). Scholars have distinguished several main approaches within the psychology of religion (35, 36, as cited in 37). Sigmund Freud, founder of psychoanalysis, saw God as a projection linked to paternal experiences, considered faith a psychopathology, and portrayed religion as a social source of restriction and suffering (38–40). Carl Jung (41), in contrast, viewed religiosity as a natural manifestation of the human psyche. For Jung, God represented an archetype—an element of the collective unconscious (42). In behaviorist theory, as represented by Skinner, religion is a product of socialization and upbringing; religious behavior results from conditioning processes (43). Humanistic approaches, though varied, share an emphasis on the subjectivity of a person’s relationship with higher beings. Maslow (44) argued that the need for self-actualization and transcendence is universal. Ideas of growth were also emphasized by Frankl (45) and Allport (46), the latter distinguishing between extrinsic and more mature, intrinsic religiosity.

The cognitive approach takes a reductionist and empirical stance on religiosity, viewing it as a product of cognitive processes and adaptation. Research in this domain seeks to understand mechanisms by studying individuals with cognitive deficits or psychiatric conditions and using advanced measurement techniques.

Both religion and religiosity are fluid concepts that can be defined from various perspectives. Lee and Early (47) noted that the lack of a single, universal definition of religiosity among psychologists stems from the complexity of the phenomenon, linguistic challenges in defining it, and the influence of personal perspectives. This lack of consensus promotes diverse and complementary theoretical and methodological approaches to the study of religion and religiosity.

Religion can be understood as a system of beliefs and practices that constitutes a social phenomenon (48–50). It can be analyzed in social, cultural, behavioral, and emotional contexts (17, 48). Religious institutions are also essential components of religion. Dubin and Graetz (12) emphasize this by describing religion as the external expression of personal faith practiced within institutional frameworks.

Religiosity refers to the individual manifestation of religion. Two central elements of religiosity are beliefs and practices proposed by a religious institution or group (48, 49, 51). This perspective emphasizes both the acceptance and internalization of beliefs and their expression through specific behaviors in private and public settings. Numerous studies have attempted to categorize religiosity by defining its dimensions. Chaim (52) provides a synthesis of commonly recognized elements that align with Glock’s (53) model. The ideological dimension reflects individual beliefs; ritual refers to religious practices; experiential relates to emotions, perceived interactions with the divine, and spiritual well-being; the intellectual dimension includes knowledge of key doctrines and sacred texts; and the consequential dimension addresses the effects of religiosity in other areas of life. Given the richness of the concept, many studies focus on specific aspects.

Factors influencing individual religiosity include family upbringing (54), socialization processes (55), and social relationships in one’s immediate environment (56). Additional factors include loneliness (57), analytical thinking style (58), intelligence (59), and perceived control (60).

The connection between religion and religiosity appears stronger than that between religiosity and spirituality. The distinction between the latter is visible in both academic discourse and public life. Spirituality is a more individual, subjective construct, often detached from institutional structures. It may focus on inner development, the search for transcendence, and life’s meaning, independent of formal religious practice. Notably, one can be religious without being spiritual and vice versa (49, 50). Because of the subjectivity involved, studying spiritual experiences empirically is particularly challenging. Given that religiosity and spirituality are not inherently linked, this article focuses specifically on the religiosity of individuals on the autism spectrum (cf. 17).

## 4 Methodology of the literature review

The aim of this review was to present the current state of knowledge on the religiosity of adults on the autism spectrum, from both theoretical and empirical perspectives. To this end, a literature review was conducted in accordance with the procedures outlined below.

### 4.1 Search criteria and databases

Literature searches were carried out using the following academic databases: Scopus, Web of Science, PsycINFO, Google Scholar, and PubMed. Keyword combinations in English included: “autism,” “autism spectrum disorder,” “ASD,” “religion,” “religiosity,” “spirituality,” “theory of mind,” “mentalizing,” and “cognitive science of religion.”

The review included publications from the years 2000–2024, with earlier, theoretically significant works also taken into account (e.g., Guthrie, 2, Baron-Cohen, 61).

### 4.2 Inclusion and exclusion criteria

Included in the review were:

- Empirical studies involving adults or adolescents (aged 12 and above) diagnosed with autism spectrum disorders;
- Works describing religiosity, spirituality, or God representations in this population;
- Peer-reviewed publications published in English or Polish.

Excluded from the review were:

- Studies focused exclusively on children (under the age of 12);
- Non-peer-reviewed works (e.g., NGO reports, blogs, essays);
- Articles that did not include religiosity as a primary focusof analysis.

### 4.3 Type of review

Given the exploratory nature of the research question and the methodological diversity of the analyzed publications, the review is narrative in nature, with elements of a systematic review. The analysis considered current and influential theoretical and empirical works, with particular emphasis on studies published in journals indexed in Scopus and Web of Science.

### 4.4 Classification of empirical studies: hypothesis-driven vs. exploratory

Two types of empirical studies were identified in the analysis:

- Hypothesis-driven studies – based on pre-establishedtheoretical models and statistically testing proposedrelationships (e.g., 10, 62, 63);
- Exploratory studies – aiming to qualitatively capture theindividual religious experiences of people with ASD withoutpredefined hypotheses (e.g., 9, 64).

This distinction enabled the identification of both general trends and subjective, nuanced religious experiences among individuals on the autism spectrum.

## 5 Religiosity in individuals on the autism spectrum

Theoretical Frameworks Explanations of the relationship between religiosity and the autism spectrum draw on various traits considered specific to autistic individuals. The most frequently cited frameworks include theory of mind and its extension—existential theory of mind. Scholars also propose hypotheses related to weak central coherence, restricted interests, cognitive rigidity, resistance to change, and mirror neuron system deficits.

### 5.1 Theory of mind and cognitive style

Researchers investigating the link between autism spectrum conditions and religiosity often refer to theory of mind (10). Developed in the late 1970s (65) and connected to autism research since the 1980s (66), theory of mind is defined as the ability to understand the mental states of others, such as beliefs, intentions, and emotions. Its core premise is the awareness that other beings (including humans and supernatural entities) possess minds distinct from our own. Numerous studies suggest that individuals on the autism spectrum may have reduced mentalizing capabilities—that is, the innate ability to apply theory of mind (61, 67).

Deeley (8) argues that mentalizing is essential for the development of religious beliefs and practices. Understanding metaphoric and symbolic messages requires the ability to interpret behavior as driven by mental states—emotions, intentions, and beliefs. This capacity is critical for grasping symbolic culture expressed in myths, narratives, and sacred texts. According to Deeley, impairments in theory of mind may result in an inability to comprehend references to religiously significant entities (8). Kéri (13) adds that deficits in theory of mind may hinder the interpretation of symbols found not only in texts but also in religious rituals.

Durbin and Graetz (12) introduced the concept of existential theory of mind, an extension of ToM that addresses why people attribute intention and meaning to accidental or tragic events (68). This framework posits the existence of a cognitive system that allows individuals to assign significance to life events and perceive them as intentional or meaningful messages. The natural human tendency to perceive intentionality can lead to belief in supernatural beings, the afterlife, or fate. People with autism may struggle to develop existential theory of mind, making it harder to interpret life events as deliberate messages from a divine source or within a broader existential framework. Instead, they may favor mechanistic explanations and avoid seeking hidden meaning. Bering (68) also hypothesizes that individuals with autism may conceptualize God more as an organizing principle or force than as a personal, intentional being capable of relationship.

McCauley et al. (10) proposed a three-stage hypothesis sequence describing the relationship between religiosity and theory of mind deficits. The first hypothesis, the social cognition content bias hypothesis, posits that understanding others’ minds facilitates the comprehension and retention of religious narratives. Building on Baron-Cohen’s (66) mindblindness theory, the second hypothesis—impaired religious understanding hypothesis—suggests that individuals on the autism spectrum experience difficulty in intuitively and creatively interpreting religious content. The final hypothesis, mind-blind atheism hypothesis, proposes that these cognitive challenges decrease the likelihood of religiosity and increase the likelihood of atheistic orientations among individuals with ASD.

A continuum model of theory of mind has also been proposed to reflect the variability of autistic individuals’ abilities in this domain (69). Similarly, Attwood (2006, as cited in Caldwell-Harris et al., 70) describes a continuum of cognitive styles that includes high-functioning autistic individuals and neurotypical individuals. It is important to note that more recent studies do not confirm a universal ToM deficit in individuals on the autism spectrum (71).

### 5.2 Weak central coherence and executive function deficits

Deeley (8) notes that individuals on the autism spectrum may struggle with central coherence—the tendency to integrate information into meaningful wholes (72). This cognitive style favors attention to detail over holistic interpretation, which may impede understanding of religious texts and practices, resulting in lower religiosity. Kéri (13) connects weak central coherence to the intense world theory, which suggests that autistic individuals experience overwhelming sensory and emotional stimuli due to neurophysiological factors. This hypersensitivity may lead to routine, restrictive behaviors and reduced participation in communal religious practices, while also fostering atypical spiritual experiences. These experiences, disconnected from social context, may result in a type of spirituality rooted in unique states of awareness rather than organized religion (13, 73).

Deeley (8) also discusses the potential combined effect of weak central coherence and executive function deficits—the latter defined as the ability to flexibly assign meaning to stimuli for adaptive purposes (74). This combination may make it harder to derive meaning from complex sets of information, including religious content, thereby reducing religiosity.

### 5.3 Restricted interests

Another hypothesis linking autism spectrum conditions to religiosity relates to the restricted interests characteristic of many autistic individuals (8). Intense preoccupation with specific topics—often unrelated to social interaction—may limit attention given to broader existential issues. The sense of meaning experienced by autistic individuals may be narrowly focused on highly specialized domains, with little reference to the self, other people, or spiritual beings. Deeley connects this to alexithymia, reduced cognitive flexibility, and diminished empathy.

### 5.4 Need for stability and cognitive rigidity

Scholars have also highlighted resistance to change and a preference for sameness as potential autism spectrum traits that influence religious beliefs and practices. The tendency toward cognitive rigidity and resistance to change may support the maintenance of unchanging beliefs, or even doctrinal religiosity (12, 13). Kéri (13) describes autistic individuals as “truth-seekers”—those who seek patterns and structure in data and systems (75). This drive may lead some to adopt religious beliefs based on logic, fixed rules, and classification systems. Combined with reduced sensitivity to intentionality—due to ToM impairments—this may foster avoidance of supernatural interpretations and preference for literal, logical reasoning.

This cognitive style also favors literal interpretations of metaphors, symbols, and figurative language (13, 76). The need for stability may extend to religious behavior as well. Dubin and Graetz (12) suggest that structured religious communities may appeal to autistic individuals due to their clarity, predictability, and defined social roles. This structure may be mirrored in religious doctrines and rituals, which tend to be regular and formulaic.

### 5.5 Broken mirror hypothesis

Some authors refer to the broken mirror hypothesis, which focuses on the role of mirror neurons (13). Mirror neurons are a type of cells located in different parts of the cerebral cortex. Their activity is observed both during the performance of a specific action and while observing another person performing the same action (77). The mirror neuron system supports the recognition of others’ action goals, imitation of actions, and the assessment of one’s own and others’ mental states (78). Mirror neurons may contribute to the development of theory of mind and complement the theory of mind system. They are considered essential for understanding others’ behaviors, imitation, and the development of social skills. They are also important in the formation of religious beliefs, as they allow for empathizing and understanding the intentions of imagined supernatural beings, and they facilitate participation in collective religious practices (13, 79). Early research confirmed mirror neuron dysfunction in individuals on the autism spectrum (80). However, this concept has faced substantial criticism and failed replications (13, 81). Contemporary hypotheses instead focus on top-down regulation of the mirror neuron system from the prefrontal cortex (13).

## 6 Religiosity in individuals on the autism spectrum – review of empirical studies

### 6.1 Mentalization, belief in god, religiosity, and religious practices

Caldwell-Harris and colleagues (70) conducted the first systematic study on religiosity in high-functioning adults with autism (HFA). The study was based on theoretical assumptions such as the autistic tendency for systematization and literal interpretation of content (12), less active intentionality detection systems linked with mentalization deficits (8), a need for stability, social discomfort, and a preference for open, welcoming religious communities (12). The authors described the cognitive style of HFA individuals as extreme and predisposed toward atheism, agnosticism, or the creation of personal belief systems. The study aimed to determine whether individuals with HFA have significantly different religious belief systems compared to neurotypical individuals.

The first method involved content analysis of forum posts discussing religion on two American online forums, one for autistic individuals and one for neurotypicals. The beliefs of 192 participants from the study group and 195 from the control group were categorized into atheism, agnosticism, personal belief systems, and theistic religions. Traits such as emphasis on rationality, literal thinking, lack of social interest, and social discomfort were coded. HFA individuals were less likely to affiliate with traditional religions and more likely to identify as atheists, agnostics, or to create personal belief systems, showing a stronger focus on rationality.

The second part was an online survey including 61 HFA individuals and 105 neurotypical controls. Data collected included autism diagnoses, current and childhood religious orientation and behaviors, and parental religious background. Questionnaires used included the Autism Spectrum Quotient (AQ), Systemizing Quotient (SQ), and Reading the Mind in the Eyes Test (RMET). Survey results supported forum findings, and a correlation was observed between AQ scores and religious beliefs. This aligns with the authors’ cognitive style continuum model. However, the study lacked control variables, and detailed data on religious behaviors and strength of beliefs were not reported. Further research was needed to verify the link between cognitive styles and religious beliefs.

Norenzayan et al. (62) conducted a study based on the premise that mentalization is a cognitive mechanism crucial for belief in a personal God, and difficulties in this area may reduce religiosity (8, 12). Three hypotheses were tested: (1) a negative relationship between autism spectrum traits and belief in God, (2) mentalization mediates this relationship, and (3) mentalization also mediates the relationship between gender and belief, potentially explaining lower religiosity in men. A small sample of teenagers from Florida, mostly male, was studied. It included 11 autistic and 13 neurotypical participants. Belief strength was assessed using four statements. Autism traits were measured with the Autism Spectrum Quotient (ASQ), and mentalization was measured indirectly using the Empathy Quotient (EQ). Parents completed two questionnaires. Controlled variables included age, gender, ethnicity, and parental education and religiosity. Results showed that autistic individuals were less likely to believe in God, and mentalization predicted belief in God. IQ was not a predictor.

Further studies were conducted with broader samples of Canadian students and American adults. The studies examined whether autism-related traits (mentalization and systemizing) and personality traits (agreeableness and conscientiousness) mediated the relationships between autism or gender and belief in God. Results confirmed a negative correlation between autism traits and belief strength. Mentalization was a mediator in both cases, while systemizing and personality traits were not. These studies validated the hypothesis that mentalization is significantly associated with belief in God, and autistic individuals tend to show weaker belief. Notably, a related study by Gervais and Norenzayan (82), which found a negative link between analytical thinking and religiosity, could not be replicated (83).

Reddish et al. (11) conducted a study on young individuals with HFA to assess whether they held different religious beliefs than neurotypical peers. Theoretical assumptions focused on mentalization deficits affecting religiosity. Seven hypotheses were tested, predicting lower belief strength in God, less anthropomorphic views of deities, lower perceived prayer effectiveness, and less preference for spontaneous prayer in autistic individuals. Controlled variables included gender, age, IQ, religious upbringing, and affiliation. Mentalization was measured using three tasks, and religiosity via questionnaires. The small sample included 19 autistic and 19 neurotypical participants in Singapore. No significant group differences were found in most religiosity dimensions, except for prayer attractiveness being higher among neurotypicals. Social skills scores correlated with prayer attractiveness. Mentalization scores differed significantly only on an advanced task. Only the less anthropomorphic deity view was correlated with lower mentalization. The study concluded that advanced mentalization does not significantly relate to religiosity. The small sample size is a limitation.

Ekblad and Oviedo (9) challenged the mentalization hypothesis. Their study investigated whether ToM limitations reduce religiosity in autistic individuals or whether developmental and cultural factors play a greater role. Their main hypothesis contradicted mainstream literature, proposing that ToM does not significantly affect autistic individuals’ religious experiences. An online survey of over 2,000 participants (38% autistic or reporting autistic traits) measured religiosity (beliefs, practices, spiritual experiences) and social functioning using the Aspie Quiz. Higher autistic traits correlated with more spiritual and paranormal experiences. Autistic individuals reported higher levels of religious practice than neurotypicals and more often created personal belief systems. These results questioned predictions of reduced religiosity due to ToM deficits and suggested that developmental and socio-cultural factors are more influential, though direct evidence was not presented. The authors advocated for exploring the diversity of autistic religiosity instead of testing pre-existing theories.

Van Ommen and Endress (84) conducted semi-structured online interviews with 13 autistic Christian adults (ages 16–55) in the UK to explore their experiences of collective religious practices. Participants had no speech impairments or learning disabilities. Thematic analysis revealed comfort-enhancing factors (community openness, service predictability) and discomfort triggers (loss of control, unexpected stimuli, social anxiety, loud music, critical sermons). Sensory hypersensitivities to smell, sound, and touch posed challenges. Respondents noted ableist liturgical texts and described unique cognitive styles marked by pattern recognition and logical inconsistencies in doctrine. Difficulties in symbolic language interpretation were noted. Autistic traits were viewed both as barriers and facilitators of religiosity. The second theme was community perception. Inclusion and awareness of autism within the faith community improved participants’ well-being, though stereotypes and invisibility of their neurodiversity remained challenges. Participants saw their traits as aiding deeper connections with God or creating barriers. The study did not assess religiosity levels but provided qualitative insight into how autistic traits affect religious engagement, supporting hypotheses on symbolic interpretation challenges (8, 13), mentalization difficulties (8, 10, 12), sensory sensitivities, and preference for stability (13). Respondents also highlighted strengths like comfort in structured communities (12) and unique cognitive styles (13).

### 6.2 The image of God in individuals on the autism spectrum

A study conducted by Schaap-Jonker et al. (63) focused on a central aspect of religiosity in individuals on the autism spectrum: their image of God. Theoretical premises included difficulties in interpreting others’ behaviors due to deficits in theory of mind, a tendency to interpret symbolic and metaphorical messages literally (8, 12), deficits in imagination, and challenges in expressing and understanding emotions. The authors hypothesized that the image of God in individuals with autism spectrum disorders (ASD) would be less reciprocal than in neurotypical individuals. It was anticipated that such an image would be associated with fewer positive feelings toward God and that God’s actions would less frequently be interpreted as supportive. It was also assumed that feelings of anxiety and inadequacy experienced in social interactions by individuals with ASD would extend into the religious context. A positive correlation was expected between the severity of autistic symptoms and the intensity of negative feelings and perceptions of God. Another hypothesis posited a relationship between cognitive rigidity and the presence of strict and dogmatic aspects in the image of God.

The study sample comprised 78 adult outpatients diagnosed with ASD residing in the Netherlands. The control group included 240 psychiatric patients without an ASD diagnosis and a nonclinical group of 459 individuals. The researchers utilized the Questionnaire God Image (QGI) to measure feelings in relation to God and perceptions of God’s actions, and the Autism Spectrum Quotient (AQ-NL) to assess the intensity of autistic traits. Religiosity was measured using a scale that determined the importance of religion to the individual. The frequency of prayer and participation in religious services were also recorded. Age and gender were controlled for, and the control groups were adjusted for religious orthodoxy, the significance of religion in the individual’s life, and the frequency of religious practices.

Findings indicated that the image of God in individuals with ASD was characterized by fewer positive attributes compared to other groups. There was also a noted decrease in positive feelings and perceived supportive actions from God. However, negative feelings did not dominate over positive ones, and faith remained significant for individuals with ASD within the Dutch population. Difficulties in social interactions correlated with fewer positive feelings toward God and predicted anxiety toward God. Interestingly, individuals with more pronounced autistic traits who declared a high importance of religion in their lives more frequently perceived God as a “judge” compared to others. The study confirmed the hypothesis that the image of God in individuals with ASD is less positive, less supportive, and associated with fewer positive feelings than in neurotypical individuals. The hypothesis of a positive relationship between the severity of autistic traits and negative perceptions of God was confirmed, as was the assumption of a more frequent occurrence of a stern and punitive image of God.

Nieuw Amerongen-Meeuse et al. (85) conducted a study examining the relationship between religiosity, ASD, anxiety disorders, and selected personality traits. The aim was to determine the extent to which God representations in patients are associated with specified mental disorders, personality traits, levels of religiosity, and psychological distress. The study distinguished between two types of anxiety related to God: anxiety due to uncertainty and anxiety caused by guilt.

The researchers recruited 103 respondents aged 17–63 from a mental health facility in Dimence, the Netherlands. Among the participants were 42 individuals diagnosed with ASD and 20 individuals diagnosed with anxiety disorders. The nonclinical group consisted of 41 individuals. The questionnaire included sections on the image of God (feelings and perceptions of God’s actions), religiosity and the importance of religion to the individual (religious salience), personality traits, and psychological distress.

Results confirmed previous findings indicating lower levels of religiosity in individuals with ASD compared to neurotypical individuals. Statistical analyses showed that among the factors considered, the image of God is primarily shaped by personality traits. An ASD diagnosis did not significantly influence the level of anxiety after accounting for the mediating roles of religiosity and distress. Personality traits commonly found in the ASD population were associated with the image of God. Low self-directedness and low reward dependence were linked to a negative image of God. The authors noted that, unlike their earlier study (63), they did not find a significantly more negative image of God among individuals with ASD compared to neurotypical individuals.

### 6.3 Supernatural experiences and relationships with invisible beings in individuals on the autism spectrum

Visuri (64) conducted an exploratory study involving interviews with 17 Swedish adolescents and young adults diagnosed with ASD. The study aimed to investigate whether individuals on the autism spectrum can establish relationships with invisible beings. Theoretical approaches related to the existential theory of mind suggest that deficits in mentalizing hinder such relationships (8, 10, 12). The author considered the roles of empathy and imagination in religious experiences involving supernatural beings.

Based on the interviews, Visuri observed that relationships with invisible beings can be significant for autistic individuals, with their experiences being coherent and meaningful. The author highlighted an imbalance between emotional and cognitive empathy in autistic individuals, manifesting as difficulty in understanding others’ emotional states. Invisible beings may be perceived as coherent and less complex, making them easier to understand and communicate with than the social environment. This may be facilitated by the absence of traditional sensory communication such as gestures and facial expressions. A pronounced tendency toward fantasizing among participants was also evident in the creation of imagined worlds based on cultural narratives like novels. Visuri noted that, in terms of mentalization, the perception of supernatural beings resembles the creation of imaginary companions. The study suggests that relationships between autistic individuals and supernatural beings can develop despite presumed difficulties arising from mentalization deficits, offering kindness, predictability, and opportunities to practice social skills in a safe environment.

Oviedo et al. (86) presented findings from two complementary studies focusing on spiritual experiences and perceptions of supernatural phenomena among adolescents and adults with ASD. The first study involved 421 neurotypical respondents aged 12–17 and 11 adolescents with ASD from the Murcia region in Spain. The authors emphasized that participants were at an age of intense religious development. Participants watched short films and read stories about supernatural events, such as miraculous healings, the presence of angels, and encounters with deceased individuals. They were then asked about the emotions these materials evoked, the extent to which they believed in their authenticity, and their religious practices.

The first study indicated similar levels of belief in the authenticity of recordings depicting supernatural events among the control group and adolescents with ASD. Individuals with ASD expressed significantly higher levels of uncertainty regarding stories involving angels compared to their neurotypical peers. The adolescents showed less interest in stories about angels than in those about contact with deceased individuals, with this difference being more pronounced among those with ASD. The authors also noted a significant correlation between religious practices and belief in supernatural events among neurotypical individuals. In contrast, among individuals with ASD, who exhibited lower levels of religiosity, interest in supernatural events was independent of religious practices.

A second similar study was conducted using the online platform Aspie Quiz as an optional survey for users. Among 318 respondents with an average age of 31, 6% were neurotypical individuals, 24% had mixed neurotypical and autistic traits, and 70% were individuals with ASD, primarily self-diagnosed. No significant differences were observed between neurotypical individuals and those on the autism spectrum regarding attitudes toward supernatural experiences. The main finding was a lower confidence in the authenticity of videos depicting angelic interventions among individuals with ASD compared to neurotypical individuals. The authors proposed two hypotheses to explain this difference. Angels may be less accepted as elements of more institutionalized religion by individuals who tend to create their own belief systems, a characteristic of some individuals with ASD. The second explanation relates to theory of mind, suggesting that the personal nature of angels may complicate understanding their intentions and actions, as well as perceiving their agency. No differences were observed in interest in supernatural phenomena between individuals with ASD and neurotypical individuals in either study. The authors hypothesized that the processes of practicing religion and learning about the spiritual experiences of close individuals may be significant factors influencing the transformation of religiosity in young people and individuals with ASD, potentially resulting in lower levels of religiosity.

## 7 Summary

Research on the religiosity of individuals on the autism spectrum has yielded mixed results, making it difficult to definitively confirm or reject the proposed hypotheses (see **Table 1**). Several studies have questioned the validity of theory of mind as a key mechanism underlying religiosity in autistic individuals (9, 11, 64). However, other findings support the hypothesis that deficits in mentalizing may contribute to lower religiosity in individuals with autism spectrum disorders (62, 70, 84, 86).

**Table 1:** Overview of selected empirical studies on the religiosity of adults on the autism spectrum by year of publication.

Authors (year)	Study type	Methodology	Sample size	Key findings
Caldwell-Harris et al. (70)	Hypothesis-driven	(1) Content analysis, (2) Survey	(1) N=192 + 195, (2) N=61 + 105	Lower affiliation with traditional religions; development of individualized belief systems
Norenzayan et al. (62)	Hypothesis-driven	Questionnaires, statistical mediation	(1) N=11 + 13 (1st study); larger samples in later studies	Lower belief in God; mentalizing ability mediates religiosity
Schaap-Jonker et al. (63)	Hypothesis-driven	QGI, AQ questionnaires	N=78 (ASD) + 459 (non-clinical) + 240 (clinical)	Less supportive image of God; more frequent perception of God as a judge
Reddish et al. (11)	Hypothesis-driven	Questionnaires, Theory of Mind tests	N=19 + 19	No significant group differences in religiosity; prayer less appealing to individuals with HFA
Ekblad & Oviedo (9)	Exploratory	Online survey	N=806 (ASD traits) + 1332 (controls)	Greater spiritual experiences and religious practice; challenges the ToM-based explanations
Visuri (64)	Exploratory	Interviews	N=17	Individuals with ASD can form meaningful and coherent relationships with supernatural beings
Oviedo et al. (86)	Exploratory	Surveys using video and textual stimuli	(1) N=11 + 421 (adolescents); (2) N=318 (adults; 70% with ASD)	Comparable interest in supernatural experiences across groups; lower trust in angelic events
van Ommen & Endress (84)	Exploratory	Semi-structured interviews	N=13	Sensory sensitivity, unpredictability, and social anxiety hinder religious participation
Nieuw Amerongen-Meeuse et al. (85)	Hypothesis-driven	Questionnaires, statistical mediation	N=42 (ASD) + 20 (anxiety) + 41 (non-clinical)	Lower religiosity in ASD; no confirmation of a more negative image of God

It remains unclear whether individuals on the autism spectrum exhibit greater (9), comparable (11, 86), or lower (62, 70, 85) levels of religiosity compared to neurotypical individuals. Similarly, findings regarding the perceived image of God and associated emotional experiences have been inconsistent 63, 85).

To date, no studies have directly tested the “broken mirror” hypothesis concerning mirror neuron dysfunction (13). Some partial evidence supports the notion that sensory hypersensitivity and cognitive rigidity may limit religiosity, while an accepting and structured religious community may promote it (84). A particularly interesting area of exploration was developed by Oviedo et al. (86) and Visuri (64), who highlighted the significant role of supernatural experiences among individuals on the autism spectrum, despite the assumed difficulties associated with theory of mind deficits (10).

**Figure 1:**
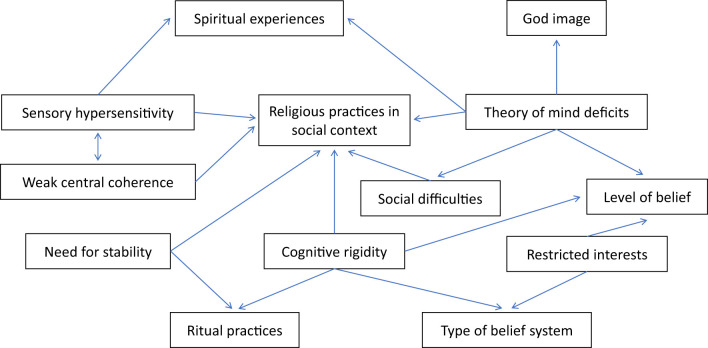
Conceptual diagram: autism traits and dimensions of religiosity.

A consistent characterization of the religiosity of individuals on the autism spectrum has not been established (63, 64, 84, 86). Similar conclusions were drawn by the authors of a review on the religiosity of adolescents on the autism spectrum (17) and by Kéri (13), who emphasized the significance of social and cultural factors. We propose a synthetic summary of the hypotheses concerning the relationships between autism-related traits and various aspects of religiosity (see **Figure 1**).

## 8 Discussion

It is worth noting that most research on the religiosity of adults on the autism spectrum has been conducted from a Western perspective. The inconsistency in results may be due to the use of various operational definitions and measurements of religiosity, autism, and mentalizing.

Given the lack of coherent findings, further research is necessary—especially studies that incorporate a broader understanding of religiosity and account for sociocultural influences. It should be emphasized that the religiosity of individuals on the autism spectrum is a complex phenomenon, shaped by many factors beyond the characteristics of autistic functioning itself. This calls for increased attention in future research to social and cultural variables, including the processes of religious upbringing and education, as well as the context of significant relationships such as family ties.

Current research predominantly reflects a Western-centric understanding of religiosity and applies measurement tools grounded in Western traditions. This results in an incomplete and potentially biased approach to the topic. It is crucial to center the perspectives of autistic individuals themselves and to apply exploratory methods when existing hypotheses are difficult to verify. Mixed-methods studies that incorporate interviews with autistic individuals may be particularly effective. Longitudinal research examining the lasting impact of sociocultural factors and the evolution of religiosity in individuals over time may also prove fruitful.

Additionally, it is essential to include participants from diverse cultural and religious backgrounds and to use tools that measure religiosity in ways appropriate to non-Western systems of belief and practice.

The present literature review also points to practical implications. The religiosity of individuals on the autism spectrum may be internally experienced and externally expressed in ways that do not align with the expectations or norms of broader society or specific social institutions such as religious communities, places of worship, educational settings, or healthcare facilities. Despite these differences, autistic individuals have religious and spiritual needs that deserve sensitive recognition. Religious leaders and community members are encouraged to cultivate openness, acceptance, and readiness to engage in dialogue and to adapt practices to accommodate the social, sensory, and cognitive needs of people on the spectrum. For religious institutions, this presents the challenge of promoting inclusivity in both practices and doctrinal communication (87).

Educators, teachers, and catechists are likewise called upon to adapt content and teaching methods in dialogue with autistic individuals and their caregivers (88). Since there is no single model of religiosity for individuals on the autism spectrum, a personalized approach is necessary—whether in religious education, communal worship, or therapeutic settings.

## Data Availability

The original contributions presented in the study are included in the article/supplementary material. Further inquiries can be directed to the corresponding author.
